# Serological Profiling of a *Candida albicans* Protein Microarray Reveals Permanent Host-Pathogen Interplay and Stage-Specific Responses during Candidemia

**DOI:** 10.1371/journal.ppat.1000827

**Published:** 2010-03-26

**Authors:** A. Brian Mochon, Jin Ye, Matthew A. Kayala, John R. Wingard, Cornelius J. Clancy, M. Hong Nguyen, Philip Felgner, Pierre Baldi, Haoping Liu

**Affiliations:** 1 Department of Biological Chemistry, University of California Irvine, Irvine, California, United States of America; 2 Department of Computer Science, University of California Irvine, Irvine, California, United States of America; 3 Institute of Genomics and Bioinformatics, University of California Irvine, Irvine, California, United States of America; 4 Department of Medicine, University of Florida College of Medicine, Gainesville, Florida, United States of America; 5 Department of Medicine, University of Pittsburgh, Pittsburgh, Pennsylvania, United States of America; 6 VA Pittsburgh Healthcare System, Pittsburgh, Pennsylvania, United States of America; 7 Department of Medicine, University of California Irvine, Irvine, California, United States of America; 8 Pacific Southwest Center for Research on Emerging Infections, University of California Irvine, Irvine, California, United States of America; Washington University School of Medicine, United States of America

## Abstract

*Candida albicans* in the immunocompetent host is a benign member of the human microbiota. Though, when host physiology is disrupted, this commensal-host interaction can degenerate and lead to an opportunistic infection. Relatively little is known regarding the dynamics of *C. albicans* colonization and pathogenesis. We developed a *C. albicans* cell surface protein microarray to profile the immunoglobulin G response during commensal colonization and candidemia. The antibody response from the sera of patients with candidemia and our negative control groups indicate that the immunocompetent host exists in permanent host-pathogen interplay with commensal *C. albicans.* This report also identifies cell surface antigens that are specific to different phases (i.e. acute, early and mid convalescence) of candidemia. We identified a set of thirteen cell surface antigens capable of distinguishing acute candidemia from healthy individuals and uninfected hospital patients with commensal colonization. Interestingly, a large proportion of these cell surface antigens are involved in either oxidative stress or drug resistance. In addition, we identified 33 antigenic proteins that are enriched in convalescent sera of the candidemia patients. Intriguingly, we found within this subset an increase in antigens associated with heme-associated iron acquisition. These findings have important implications for the mechanisms of *C. albicans* colonization as well as the development of systemic infection.

## Author Summary

*Candida albicans* has both a benign and pathogenic association with the human host. Previous to this study, little was known in regard to how the host humoral system responds to the commensal colonization of *C. albicans*, as well as the development of hematogenously disseminated candidiasis. We show using a *C. albicans* cell surface protein microarray that the immunocompetent host exists in permanent host-pathogen interplay with commensal *C. albicans,* and undergoes stage-specific antibody responses as the yeast transitions from a benign microbe to an opportunistic fungal pathogen. Also identified were serological signatures specific for acute and convalescent stages of candidemia. Our findings provide new insight in the characterization of potential serodiagnostic antigens and vaccine candidates to the opportunistic pathogen *C. albicans*.

## Introduction

The yeast *Candida albicans* exists in a dichotomist relationship with the human host. *C. albicans* is frequently found as a commensal organism on the human skin, gastrointestinal (GI) tract and the vulvovaginal tract [1]. Close to 60% of healthy individuals carry *C. albicans* as a commensal in the oral cavity. Colonic and rectal colonization is even higher, ranging from 45% to 75% among patient groups. Alterations in the host immunity, physiology, or normal microflora rather than the acquisition of novel or hypervirulent factors associated with *C. albicans*, are suggested to lead to the development of candidiasis [2]. Both neutrophils and mucosal integrity of the GI tract, are critical in preventing hematogenously disseminated candidiasis [3]. The development of candidemia can begin with the translocation of *C. albicans* into the bloodstream from initial commensal GI colonization or the shedding from developing biofilms on indwelling catheters [4],[5]. Fungal cells that evade the host immune system can spread to deep organ systems leading to hematogenously disseminated candidiasis, which has an estimated mortality rate of 40%, even with the use of antifungal drugs [2].

Information on *in vivo* gene expression would provide insight into how *C. albicans* interacts with host cells during the transition from commensal colonization to an opportunistic pathogen in the immunocompromised host. However, *in vivo* transcription profiling of *C. albicans* during commensal colonization or candidemia is technically challenging [6]. Instead, several genome-wide transcriptional analyses of *C. albicans* responses to host cells have been performed using *ex vivo* and *in vivo* infection models. These include phagocytosis of *C. albicans* cells by neutrophils [7] and macrophages [8], exposure to human blood, plasma, and blood cells [9],[10], as well as invasion of perfused pig liver and reconstituted human epithelium [11],[12]. Genes that are associated with morphological changes, metabolic adaptation, and oxidative stress are the major responses of *C. albicans* to host cells identified in these studies. The changes in gene expression identified in these *in vitro* model systems possibly reflect tissue- or stage-specific expression during an infection in patients. Profiling of antibody responses during infection in patients offers an alternative approach that can overcome technical challenges of *in vivo* transcription profiling. An antibody-based approach has been used to identify *C. albicans* gene expression during thrush in individuals with HIV [13].

Currently the isolation of *C. albicans* from blood cultures is the standard method for the diagnosis of candidemia. Nevertheless, blood cultures may only become positive late in infection, and in one study up to 50% of all autopsy-proven cases of candidemia were reported as negative in blood cultures [14]. Thus, the ability to rapidly and easily diagnose candidiasis is urgently needed. An alternative approach to microbiological confirmation of *C. albicans* infection is serological diagnosis. An immunoproteomic approach using two-dimensional electrophoresis followed by quantitative Western blotting and mass spectrometry has been used to profile serologic response to peptides from cell surface extracts in candidemia [15]–[17]. A significant proportion of antigens identified were glycolytic enzymes and heat shock proteins. An antigenic multiplex consisting of the peptides Bgl2, Eno1, Pgk1, Met6, Gap1, and Fba1 provides 87% sensitivity and 74% specificity when distinguishing patients with candidemia from uninfected hospital patients [17]. However, this approach has several limitations; only the most abundant and soluble proteins can be resolved on the immunoblot, there is a lack of reproducibility of cell wall preparations, and most importantly, there is the inability to account for various stage- and tissue-specific gene expressions from the cultured cells. These limitations can be addressed by using a protein microarray to profile antibody responses [18]–[21].

To investigate the establishment of the humoral immunity during commensal sensitization, as well as the adaptive immune response to candidemia, we have developed a *C. albicans* cell surface protein microarray. Our rationale in developing a cell surface protein microarray is that the cell surface of *C. albicans* is the immediate target of the human immune system when *C. albicans* cells enter the bloodstream. Cell surface proteins play important roles in host interaction, and many of them are known virulence factors. In addition, a recent study showed that there is a significant expansion of cell wall, secreted and transporter gene families in pathogenic *Candida* species in comparison to non-pathogenic yeasts [22]. In this study, profiling of serological response on the protein microarray with sera from candidemia patients, blood-culture negative hospital patients and healthy individuals lead to the identification of serological signature specific for acute and convalescent stages of candidemia. Intriguingly, large proportions of the identified antigens are involved in oxidative stress, drug resistance and iron acquisition. Furthermore, strong IgG response to many proteins known to be induced and/or required for *C. albicans* invasion of epithelial and endothelial cells is observed in both candidemia patients and non-candidemia controls, including all healthy individuals. Our findings provide new insights into commensal colonization and pathogenesis of *C. albicans*, as well as the characterization of potential serodiagnostic antigens and vaccine candidates.

## Results

### Sera collection and study population

Hospital patient sera were collected from Shands Hospital at the University of Florida (UF) (SH-UF) from January 2004 to December 2006. We collected sera from 21 patients with candidemia where the etiological agent was *C. albicans*. The median time from the date of positive culture to serum collection was two days. The study population was classified by age, gender, underlying disease, portal of entry, antifungal received, and outcome of stay (Table S1). A subset of the candidemia patients was followed through acute infection (days 0–14) to early convalescent (week 4) and mid convalescent (week 12) infection. We also used sera from 12 hospital patients and 50 healthy individuals who had no evidence of candidiasis as our negative control groups.

### *C. albicans* cell surface protein microarray construction and hybridization

*C. albicans* cell surface proteins were chosen for the protein microarray because they interact directly with the host and thus are likely important for colonization and infection, as well as likely targets for the host immune system. Furthermore, many of their protein expression levels are regulated in response to extracelluar signals, such as stress, nutrients, host factors, or changes in environment. Known antigenic proteins are also included as controls (Bgl2, Eno1, Pgk1, Gap1, Cdc19, Tkl1, Hsp90, and members of the Hsp70 family) [15],[17]. The collection contains 451 His- and HA-tagged peptides (Table S2) that represent 363 different proteins, since ORFs >3,000 bps were cloned into two or more segments. All tagged proteins were confirmed individually by western blot and again on the protein microarray.

We have used the *C. albicans* cell surface microarray to evaluate the antibody profile of patients with candidemia against healthy individuals and uninfected hospital patients to determine relevant cell surface antigens that correlate with infection. Arrays were probed with a collection of sera consisting of different stages of candidemia: acute, early convalescent (approximately 4 weeks after onset of infection) and mid convalescent (approximately 12 weeks after onset of infection), as well as uninfected hospital patients and healthy individuals. Figure S1 shows a representative image of the microarray hybridized with the serum of an acute candidemia patient. All hybridizations in this study were done under the same conditions and dilutions with protein microarrays printed from the same batch. Their serological reactivity is shown as a heatmap where the antigens are sorted by increasing normalized global mean intensity, with bright green having the weakest intensity, red being the strongest, and black in between (Figure S2). An examination of the IgG response to the entire *C. albicans* cell surface protein microarray showed that the mean global signal intensity was similar among different groups (data not shown), although antigenic profiles are not identical between individuals.

### Characterization of an IgG response indicative of permanent host-pathogen interplay in commensal colonization

**Figure 1:**
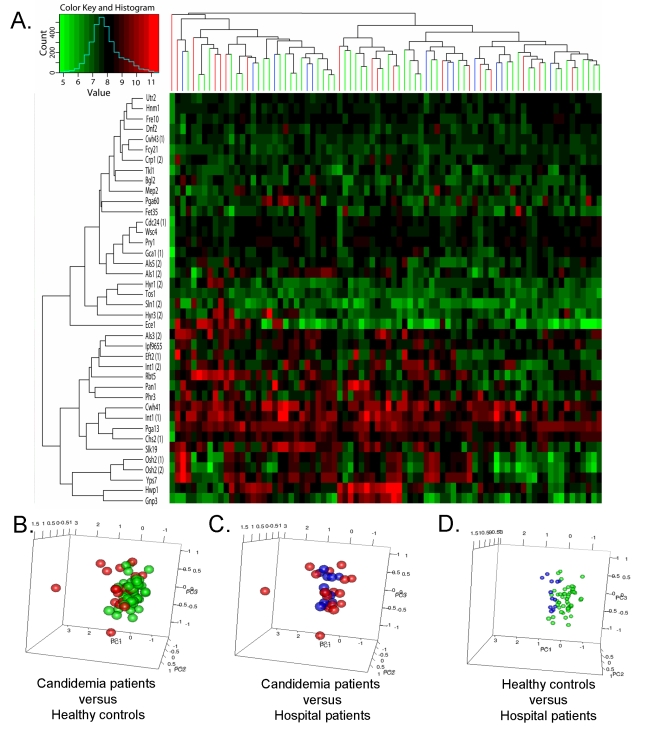
Prevalence of the serodominant anti-*C. albicans* cell surface IgGs in the study population. (A) Two-way hierarchical cluster of the top-forty serodominant antibodies (rows) and serum specimens (columns) from acute candidemia patients (n = 18) and negative controls groups (hospital patients (n = 12) and healthy individuals (n = 50)). The color scale ranks the antigens with red being the strongest, bright green the weakest, and black in between. (B, C & D) Principal component analyses of top-forty serodominant anti-*C. albicans* cell surface IgG antibody expression profiles between acute candidemia patients and each negative control group (hospital patients and health individuals). Each circle denotes the anti-*C. albicans* cell surface antibody profile of a single serum specimen. Samples are color coded as the following acute candidemia patients (red), healthy individuals (green), and hospital patients (blue).

We were interested in determining the most seroprevalent antibodies in the acute candidemia patients and how their humoral response compared against the negative control groups. Antigens to the most seroprevalent antibodies were defined as serodominant antigens and characterized as having mean antigen reactivity 2-fold greater than the *in vitro* transcription/translation reaction mixture containing no vector. The top-forty serodominant antigens in the candidemia patients consisted of many previously characterized antigenic peptides such as Bgl2 [17], Tkl1 [15], Hwp1 [13],[23], Eft2 [15], and Cdc24 [13] (Table 1). Also among the top-forty serodominant antigens were many previously identified virulence-associated and/or hyphal-regulated proteins (eg. Int1, Hwp1, Als1, Als3, Als5, Ece1, Hyr1, Cdc24, and Utr2) (Table 1) [24]–[32]. Interestingly, this serological response of acute candidemia patients was shared with both uninfected hospital patients and healthy individuals. The mean signal intensity to the top-forty serodominant antigens was 8,825 in acute candidemia patients, 8,837 in uninfected hospital patients, and 10,790 in healthy individuals. A two-way hierarchical cluster analysis of the top-forty serodominant antigens shows that the serum specimens of both the positive and negative candidemia groups were randomly dispersed throughout the hierarchical tree (Figure 1A). To further confirm that the top-forty serodominant antigenic signatures are shared among acute candidemia patients, the uninfected hospital patients and healthy individuals, principal component analysis (PCA) was used to generate a three-dimensional projection of the data (Figure 1B, 1C and 1D). The PCA shows that a large proportion of both the positive and negative acute candidemia sera are clustered together. These analyses suggest that IgG levels to the top-forty serodominant antigens are similar in both the negative control groups and acute candidemia sera. Since many of the top-forty antigens are either important for or induced during the invasion of epithelial or endothelial cells [11],[33], their expression in healthy people, inferred from the presence of their antibodies, indicates the existence of a permanent host-pathogen interplay in immunocompetent individuals.

**Table 1:** The forty most serodominant antigens in acute candidemia patients.

Name		Description	Mean antigen reactivity (+/− SEM)		BH adjusted p-values
Systematic	Common		Candidemia patients	Negative controls	
19.4257 (1)	Int1 (1)	Integrin-like protein	35,380 (11,412–109,691)	32,163 (12,268–84,323)	0.853
19.4421	Cwh41	Glucosidase	32,758 (13,701–78,323)	40,288 (16,253–99,864)	0.642
19.6420	Pga13	Unknown function	27,057 (10,402–70,383)	40,478 (25,960–63,115)	0.116
19.5636	Rbt5	Hemoglobin utilization	19,351 (4,882–76,695)	12,787 (3,956 - 41,338)	0.419
19.1321	Hwp1	Hyphal wall protein	19,136 (4,604–79,530)	15,974 (4,407–57,899)	0.776
19.6763	Slk19	Unknown function	18,546 (6,904–49,823)	15,590 (4,535–53,590)	0.769
19.6481	Yps7	Aspartic-type peptidase	14,170 (4,202–47,787)	15,103 (4,189–54,451)	0.915
19.1816 (2)	Als3 (2)	Agglutinin-like protein; iron assimilation	13,292 (5,047–35,009)	16,960 (7,384–38,953)	0.531
19.7298 (1)	Chs2 (1)	Chitin synthase	12,221 (5,271–28,335)	20,418 (13,475–30,940)	0.0361
19.5788 (1)	Eft2 (1)	Elongation Factor 2	11,973 (3,530–40,612)	9,139 (3,878–21,538)	0.504
19.3988	Ipf9655	Unknown function	11,642 (5,339–25,386)	12,904 (6,096–27,318)	0.806
19.7565	Gnp3	Glutamine permease	8,919 (2,049–38,826)	6,306 (1,686–23,592)	0.535
19.5632	Phr3	Glucanosyltransferase	8,845 (2,542–30,768)	12,600 (4,498–35,298)	0.436
19.3374	Ece1	Unknown function	8,571 (1,967–37,354)	3,401 (702–16,481)	0.105
19.4565	Bgl2	Glucanosyltransferase	8,437 (4,642–15,334)	5,564 (3,022–10,244)	0.116
19.886	Pan1	Actin cytoskeleton-regulatory complex	8,368 (2,924–23,953)	14,845 (5,032–43,796)	0.177
19.4257(2)	Int1 (2)	Integrin-like protein	8,256 (2,986–22,828)	12,856 (4,405–37,517)	0.321
19.5095 (2)	Osh2 (2)	Oxysterol-binding protein	8,103 (2,696–24,359)	6,953 (1,832–26,385)	0.806
19.5095 (1)	Osh2 (1)	Oxysterol-binding protein	8,070 (2,178–29,899)	10,254 (3,080–34,142)	0.667
19.4784 (2)	Crp1 (2)	Copper transporter	7,937 (3,951–15,943)	7,260 (4,836–10,899	0.751
19.2787	Pry1	Unknown function	7,813 (4,172–14,634)	13,674 (9,461–19,764)	0.00451
19.5588	Pga60	Unknown function	7,477 (4,350–12,852)	8,332 (3,111–22,311)	0.808
19.1671	Utr2	Glycosidase	7,466 (4,590–12,144)	8,456 (6,440–11,102)	0.479
19.2003	Hnm1	Choline transporter	7,385 (4,798–11,367)	8,333 (6,454–10,759)	0.470
19.4975 (2)	Hyr1 (2)	Hyphal wall protein	7,221 (3,177–16,411)	4,665 (2,527–8,610)	0.0814
19.7251	Wsc4	Unknown function	6,906 (3,926–12,150)	9,603 (7,001–13,172)	0.0457
19.3174 (1)	Cdc24 (1)	GDP-GTP exchange factor	6,888 (3,544–13,387)	10,759 (7,607–15,215)	0.0106
19.575 (2)	Hyr3 (2)	Unknown function	6,846 (2,378–19,712)	5,695 (2,069–15,676)	0.688
19.932	Dnf2	Phospholipid translocase	6,766 (4,655–9,836)	7,475 (4,596–12,156)	0.688
19.5672	Mep2	Ammonium permease	6,743 (3,949–11,514)	7,885 (3,644–17,059)	0.652
19.4899 (1)	Gca1 (1)	Glucoamylase	6,640 (2,881–15,302)	9,427 (5,866–15,151)	0.116
19.3225 (1)	Cwh43 (1)	Unknown function	6,537 (4,850–8,809)	6,671 (4,821–9,231)	0.921
19.1415	Fre10	Ferric reductase	6,528 (4,408–9,667)	9,450 (6,428–13,893)	0.0214
19.5736 (2)	Als5 (2)	Agglutinin-like protein	6,231 (2,848–13,634)	7,448 (3,933–14,103)	0.556
19.5741 (2)	Als1 (2)	Agglutinin-like protein	5,795 (2,489–13,495)	10,653 (4,371–25,966)	0.0649
19.3256 (2)	Sln1 (2)	Histidine kinase; osmosensor	5,561(1,535–20,139)	2,866 (1,695–4,846)	0.00878
19.1357	Fcy21	Purine-cytosine permease	5,445 (3,827–7,748)	5,895 (4,377–7,937)	0.678
19.1690	Tos1	α-agglutinin anchor subunit	5,412 (3,340–8,768)	3,994 (2,535–6,291)	0.116
19.4215	Fet34	Multicopper ferroxidase	5,372 (2,502–11,534)	6,232 (2,745–14,147)	0.689
19.5112	Tkl1	Transketolase	5,337 (2,756–10,335)	7,143 (3,938–12,957)	0.261

### Identification of antigens correlative with the acute-stage of candidemia

To determine stage-specific biomarkers of acute candidemia, the normalized serological expression of acute candidemia patients were compared against the humoral reactivity of the uninfected hospital patients and healthy individuals. Serodiagnostic antigens were defined as having an IgG response significantly greater in acute candidemia patients (days 0–14) as compared to the negative control groups with Benjamini and Hochberg (BH) adjusted Cyber-T p-values <0.05. Thirteen antigens met this requirement (Table 2). Moreover, among the proteins identified as serodiagnostic markers, proteins involved in oxidative stress response appeared to be enriched over other functional categories. Sln1 and Nik1 are two out of three histidine kinases on the cell surface protein microarray and they are both identified as serodiagnostic antigens. Sln1 and Nik1 are sensors for the high-osmolarity glycerol (HOG) pathway, a mitogen-activated protein kinase cascade responsible for osmotic and oxidative stress adaptation in *C. albicans* [34],[35]. In addition, the expression levels of *CDR4, RAS2,* and *ALS9* are up-regulated during oxidative stress [35]. Another functional group over-represented among the serodiagnostic antigens are transporters associated with drug resistance (Cdr1, Cdr4, and Yor1) [36].

**Table 2:** Antigenic biomarkers of the acute candidemia patients.

Name		Description	Mean antigen reactivity (+/- SEM)		BH adjusted p-value	AUC
Systematic	Common		Candidemia patients	Negative controls		
19.6000 (3)	Cdr1 (3)	Drug transporter	2,842 (1,295–6,234)	837 (440–1,593)	1.04E-07	0.873
19.1844	Cfl91	Ferric reductase	3,522 (778–15,943)	752 (329–1,719)	1.47E-06	0.792
19.5079 (3)	Cdr4 (3)	Drug transporter	4,433 (2,104–9,341)	2,089 (1,285–3,394)	1.07E-04	0.777
19.5742 (2)	Als9 (2)	Agglutinin-like protein	4,233 (2,167–8,267)	2,025 (1,037–3,956)	1.96E-03	0.786
19.3575	Cdc19	Pyruvate kinase	3,235 (1,364–7,673)	1,704 (960–3,024)	6.10E-03	0.755
19.5181 (2)	Nik1 (2)	Osmosensor	2,420 (975 – 6,008)	1,198 (605–2,372)	6.51E-03	0.722
19.5384 (2)	Chs8 (2)	Chitin synthase	2,306 (984–5,402)	1,267 (739–2,170)	6.69E-03	0.750
19.6595	Rta4	Phospholipid transporter	3,011 (1,802–5,030)	1,506 (690–3,285)	6.94E-03	0.764
19.3256 (2)	Sln1 (2)	Osmosensor	5,561 (1,535–20,139)	2,866 (1,695–4,846)	8.78E-03	0.630
19.600 (2)	Trk1 (2)	Potassium transporter	2,780 (1,483–5,211)	1,652 (890–3,066)	0.0214	0.784
19.1783 (3)	Yor1 (3)	Drug transporter	2,566 (1,024–6,427)	1,593 (989–2,565)	0.0269	0.651
19.6926	Csc25	Guanyl-nucleotide exchange factor	2,507 (1,710–3,675)	1,563 (834–2,930)	0.0362	0.735
19.5902	Ras2	RAS signal transduction	3,005 (1,872–4,824)	2,032 (1,234–3,348)	0.0417	0.704

**Figure 2:**
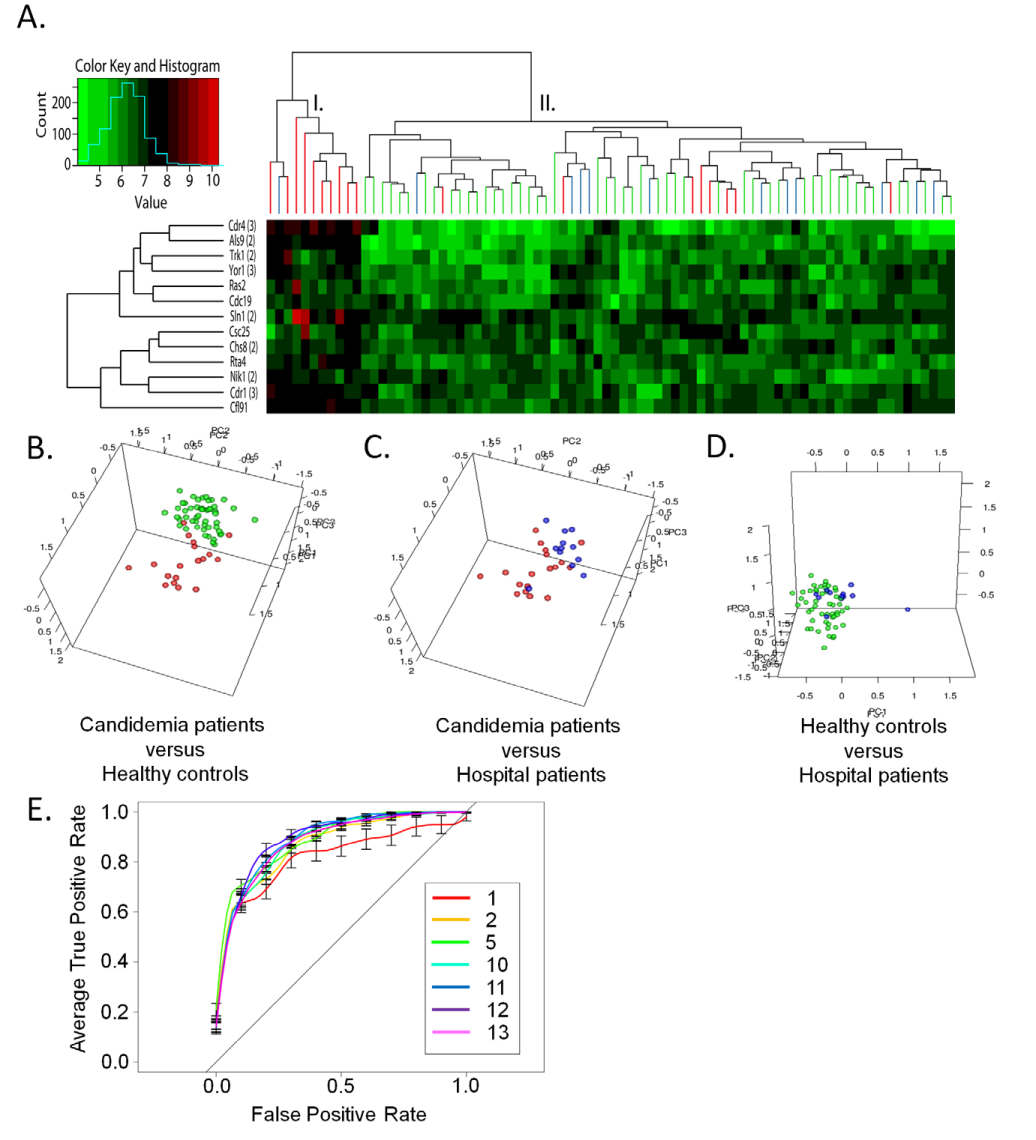
Discrimination of acute candidemia patients from the study population. (A) Two-way hierarchical cluster analyses of the 13 differentially expressed anti-*C. albicans* cell surface antibodies in acute candidemia sera. The heatmap is organized with antigens, in rows, and acute candidemia patients (n = 18) and negative controls groups (hospital patients (n = 12) and healthy individuals (n = 50)) in columns. The colorized scale ranks the antigens with red being the strongest, bright green the weakest, and black in between. (B, C & D) Principal component analyses of serum anti-*C. albicans* cell surface IgG antibody expression profiles that discriminate between candidemia patients and each negative control group (hospital patients and health individuals). Each circle denotes the anti-*C. albicans* cell surface antibody profile of a single serum specimen. Samples are color coded as the following acute candidemia patients (red), healthy individuals (green), and hospital patients (blue). (E) The graph shows the ROC curves generated using different sets of acute serodiagnositc antigens.

The 13-serodiagnostic antigens were also evaluated with a two-way hierarchical cluster analysis on candidemia positive and negative sera. Interestingly, the sera clustered into two distinct groups based on their responses to the 13 antigens (Figure 2A). Cluster I contained 10 candidemia sera and only one uninfected hospital patient. Cluster II contained all 50 healthy individuals, 11 of the 12 hospital patients, and 8 acute candidemia sera (Figure 2A). To further confirm that the antigenic signatures identified during the acute phase of candidemia differed from the negative control groups, PCA was used to create a three-dimensional projection of the data (Figure 2B, 2C, and 2D). In agreement with the two-way hierarchical cluster analysis, two distinct groups were observed (Figure 2B and 2C). Also, the PCA of the negative control groups showed individuals are clustered together with the exception of one outlying uninfected hospital patient found clustered with the acute candidemia patients (Figure 2C and 2D). These data provide further support of the antigenic signature of patients during the acute phase of candidemia. Multiple linear regression models determined that the antigenic profiles of acute candidemia patients were not related to various risk factors (i.e. age, gender, course of treatment, coexisting disease, and recovery/fatality) (data not shown). However, this determination is limited by the small sample size of our study.

Multiple independent serodiagnostic antigens can dramatically improve the sensitivity and accuracy of serodiagnostic tests [37]. To establish a collection of antigens that could be used as a multiplex set to accurately distinguish candidemia cases from controls, we studied the discriminatory power of different sets of proteins using receiver operating characteristic (ROC) curves. First, ROC curves were generated for individual serodiagnostic antigens and the area under the ROC curves (AUC) for each antigen is listed in Table 2. The top-five cell surface proteins all have an AUC greater than 0.76, with CDR1 (3) (AUC 0.87, BH adjusted Cyber-T p-value <1.04e-7) giving the best single antigen discrimination (Table 2). The 13^th^ antigen has an AUC of 0.630, which still exceeds the upper 95% confidence interval for random expectations for the AUC. To extend the analysis to combinations of antigens, we used kernel methods and support vector machines to build linear and nonlinear classifiers. As inputs to the classifier, we used the highest-ranking AUC antigens in combinations of 2, 5, 10, 11, 12 and 13 proteins and the results were validated with 10 runs of three-fold cross-validation (Figure 2E). Increasing the antigen number from 2 to 5, and 5 to 10 produced improvements in the classifier. But as the antigens increased to 13, a reduction in accuracy was observed. Using the ten most significant diagnostic antigens (in rank order: Cdr1 (3), Cfl91, Cdr4 (3), Als9 (2), Cdc19, Nik1 (2), Chs8 (2), Rta4, Sln1 (2), and Trk1 (2)), the classifier predicts 83% (95% CI, 76–89%) sensitivity, 72% (95% CI, 68–76%) specificity, and 74% (95% CI, 72–76%) accuracy in diagnosis of acute phase candidemia from the negative controls (healthy individuals and uninfected hospital patients) (Table 3).

**Table 3:** Test operating characteristics of the clinical biomarkers.

	Percentage (CI 95%)		
*C. albicans* clinical biomarkers	Sensitivity	Specificity	Accuracy
Acute phase^1^	83 (76–89)	72 (68–76)	74 (72–76)
Convalescent phase^2^	93 (89–96)	96 (95–96)	95 (94–96)

1 On the basis of two-way hierarchical cluster results of the top ten differentially expressed anti-*C. albicans* cell surface IgG antibodies from acute phase (days 0-14) of candidemia patients and negative control groups (i.e. healthy individuals and hospital patients).

2 On the basis of two-way hierarchical cluster results of the top five differentially expressed anti-*C. albicans* cell surface IgG antibodies from early (week 4) and mid (week 12) convalescent phases of candidemia patients and negative control groups (i.e. healthy individuals and hospital patients).

### Identification of antigens correlative with the convalescent-stage of candidemia

We were next interested in identifying antigens that are significantly different between the early/mid convalescent candidemia patients (weeks 4 and 12 of the infection, respectively) and the negative control groups. The convalescent patient sera consisted of three patients whose serum was drawn under all three disease phases (acute phase, early and mid convalescent phases), 4 patients who had blood drawn at the acute and early convalescent phases, and 3 patients whose blood was drawn only at the early convalescent phase. Using BH adjusted Cyber-T p-values <0.05, we identified 33 antigens, 11 of which are from the 13 diagnostic antigens for the acute phase of infection (Table 4). Among the identified convalescent biomarkers were marked expansions in proteins involved in iron acquisition (Rbt5, Csa1, Flc1, and Cfl91) (Table 4). Cfl91 is a putative ferric reductase similar to Fre10, which is required for the release of iron from transferrin and the reduction to ferrous iron [38]. The protein Flc1 has been identified as having heme uptake activity [39] whereas, both Rbt5 and Csa1 have been implicated as receptors of hemoglobin whose function is to deliver the hemoglobin by endocystosis to the vacuole where iron is released by acidification [40],[41]. The remainders of the identified proteins have roles in cell wall biogenesis, membrane lipid organization, and drug resistance.

**Table 4:** Antigenic biomarkers of the early and mid convalescent candidemia patients.

Name		Description	Mean antigen reactivity (+/- SEM)		BH adjusted p-value	AUC
Systematic	Common		Candidemia patients	Negative controls		
19.1844	Cfl91	Ferric reductase	10,699 (4,436–25,802)	752 (329–1,719)	0	0.969
19.323 (2)	Drs23 (2)	Phospholipid translocase	3,249 (1,812–5,826)	707 (439–1,137)	4.21E-14	0.960
19.5079 (3)	Cdr4 (3)	Drug transporter	9,831 (5,588–17,295)	2,089 (1,285–3,394)	2.28E-13	0.957
19.2296 (2)	Ipf25023 (2)	Unknown function	3,128 (1,446–6,763)	461 (234–908)	3.46E-13	0.945
19.1783 (3)	Yor1 (3)	Drug transporter	6,719 (3,759–12,009)	1,593 (989–2,565)	6.91E-13	0.970
19.6000 (3)	Cdr1 (3)	Drug transporter	3,635 (2,139–6,178)	837 (440–1,593)	1.00E-09	0.964
19.7414 (2)	Als6 (2)	Agglutinin-like protein	2,868 (1,176–6,990)	883 (569–1,372)	4.56E-09	0.917
19.1800	Vps62	Unknown function	19,259 (10,436–35,543)	5,032 (2,139–11,841)	1.33E-05	0.896
19.5759 (3)	Snq2 (3)	Drug transporter	2,580 (1,740–3,825)	1,313 (918–1,877)	1.82E-05	0.896
19.5742 (2)	Als9 (2)	Agglutinin-like protein	5,504 (3,654–8,290)	2,025 (1,037–3,956)	3.14E-05	0.913
19.5636	Rbt5	Hemoglobin utilization	67,414 (30,441–149,296)	12,787 (3,956–41,338)	5.45E-05	0.878
19.600 (2)	Trk1 (2)	Potassium transporter	4,189 (2,131–8,233)	1,652 (890–3,066)	1.07E-04	0.922
19.4565	Bgl2	Glucanosyltransferase	13,462 (7,309–24,793)	5,564 (3,022–10,244)	2.53E-04	0.866
19.5181 (2)	Nik1 (2)	Osmosensor	3,090 (1,075–8,878)	1,198 (605–2,372)	4.58E-04	0.945
19.6595	Rta4	Phospholipid transporter	3,847 (2,330–6,354)	1,506 (690–3,285)	4.99E-04	0.837
19.5384 (2)	Chs8 (2)	Chitin synthase	2,551 (1,650–3,942)	1,267 (739–2,170)	6.32E-04	0.841
19.7214	Ipf885	Glucosidase	2,948 (1,260–6,898)	1,308 (675–2,534)	1.58E-03	0.764
19.4015	Cag1	α-subunit of heterotrimeric G protein	7,808 (3,381–18,032)	4,380 (3,029–6,334)	1.58E-03	0.761
19.2946	Hnm4	Choline permease	3,001 (1,378–6,537)	1,251 (580–2,697)	1.76E-03	0.775
19.6861 (2)	Apc5 (2)	Subunit of Anaphase-Promoting Complex	4,529 (2,365–8,674)	2,054 (1,022–4,129)	2.00E-03	0.793
19.3256 (2)	Sln1 (2)	Osmosensor	6,043 (1,635–22,332)	2,866 (1,695–4,846)	2.99E-03	0.639
19.6515	Hsp90	Heat shock protein	6,188 (1,263–30,307)	2,652 (1,481–4,748)	3.06E-03	0.620
19.3575	Cdc19	Pyruvate kinase	3,613 (1,033–12,639)	1,704 (960–3,024)	3.44E-03	0.706
19.7114 (2)	Csa1 (2)	Hemoglobin utilization	9,329 (4,016–21,671)	2,713 (792–9,293)	3.71E-03	0.798
19.3269 (2)	Gsl2 (2)	Glucan synthase	3,898 (2,725–5,575)	2,418 (1,561–3,746)	6.80E-03	0.806
19.4035	Pga4	Glucanosyltransferase	3,909 (2,496–6,124)	2,326 (1,297–4,170)	0.0203	0.774
19.2501 (2)	Flc1 (2)	Heme transporter	2,565 (1,564–4,208)	1,620 (1,004–2,615)	0.0220	0.774
19.7298 (2)	Chs1 (2)	Chitin synthase	2,504 (1,660–4,208)	1,529 (855–2,733)	0.0346	0.752
19.4940	Ipf22247	Histidine permease	4,801(2,852–8,081)	3,096 (1,887–5,078)	0.0391	0.730
19.2222	Yck22	Unknown function	4,387 (3,338–5,767)	2,913 (1,795–4,728)	0.0391	0.795
19.7313	Ssu1	Sulfite transporter	3,753 (2,853–4,936)	2,503 (1,533–4,085)	0.0405	0.775
19.1648 (1)	Rad50 (1)	DNA double strand break repair	6,226 (2,722–14,237)	3,323 (1,572–7,024)	0.0405	0.715
19.5148 (2)	Cyr1 (2)	Adenylyl cyclase	3,042 (2,240–4,130)	1,880 (1,002–3,526)	0.0478	0.747

**Figure 3:**
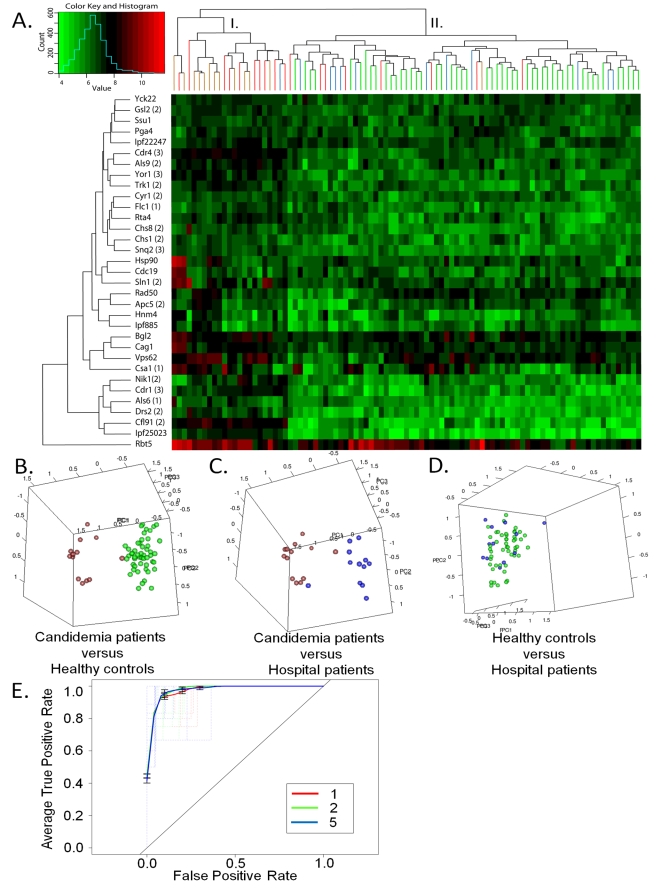
Discrimination of convalescent candidemia patients from the study population. (A) Two-way hierarchical cluster analyses of the 33 differentially expressed anti-*C. albicans* cell surface antibodies from early/mid convalescent candidemia sera. The heatmap is organized with antigens, in rows, and acute candidemia patients (n = 18), early and mid convalescent patients (n = 10) and negative control groups (hospital patients (n = 12) and healthy individuals (n = 50)) in columns. The colorized scale ranks the antigens with red being the strongest, bright green the weakest, and black in between. (B, C & D) Principal component analyses of serum anti-*C. albicans* cell surface IgG antibody expression profiles that discriminate between convalescent candidemia patients and each negative control group (hospital patients and health individuals). Each circle denotes the anti-*C. albicans* cell surface antibody profile of asingle serum specimen. Samples are color coded as the following acute candidemia patients (red), convalescent candidemia patients (brown), healthy individuals (green), and hospital patients (blue). (E) The graph shows the ROC curves generated using different sets of serodiagnostic antigens.

We next evaluated antibody response to the 33 antigens in the acute, convalescent candidemia patients and the negative control groups by two-way hierarchical cluster analysis. The individuals in Cluster II were the same as those identified previously with 13 serodiagnostic antigens (Figure 2A and 3A) with the addition of one convalescent candidemia patient whose only sera was drawn during week 4 of the infection. Individuals in Cluster I consisted of candidemia patients with the exception of the one uninfected hospital patient from Figure 2A. Three of the candidemia patients' acute and convalescent profiles were all found in Cluster I, whereas four candidemia patients' profiles converted from Cluster II to I during the convalescence phase of the disease. In addition, the remaining two-candidemia patients whose only blood draws were during week 4 also grouped in Cluster I (Figure 3A). This conversion of the antigenic profile from the negative control groups (Cluster II) to the antigenic profile consistent with candidemia (Cluster I), indicates an adaptive immune response to *C. albicans* that is different from commensal sensitization. Again, PCA was used to further confirm that the antigenic signatures identified during the convalescent phase of candidemia differed from the negative control groups (Figure 3B, 3C and 3D). ROC curves were generated to assess the ability to separate the control and convalescent candidemia. AUC was determined for each of the 33-serodiagnostic antigens and listed in Table 4 in decreasing order. The top-five ORFs all have an AUC greater than 0.94. We then used SVMs to build multiplex classifiers with 2, 5, and 10 antigens with the highest-ranking AUC from Table 4. The results were validated with 10 runs of three-fold cross-validation (Figure 3E). Increasing the antigen number from 2 to 5 maintained the diagnostic accuracy in the classifier and a reduction in accuracy occurred as the antigens increased to 10 due to over-fitting. The top-five serodiagnostic antigens are associated with xenobiotic-transporting activity (Cdr4 and Yor1) [36], phospholipid-transporting activity (Drs23), a putative ferric reductase (Cfl91), and a mucin-like cell wall protein (Ipf25023) (Table 4). Using the top-five antigens, the classifier predicts 93% (95% CI, 89–96%) sensitivity, 96% (95% CI, 95–96%) specificity, and 95% (95% CI, 94–96%) accuracy in the differentiation of early/mid convalescent phase candidemia from the negative controls (healthy individuals and uninfected hospital patients) (Table 3).

Having identified 33 antigens that are correlative with convalescent candidemia in comparison to the negative control groups, we next wanted to determine the temporal change in IgG response to these 33 antigens during the transition from acute infection (AI), to early convalescent (EC), and mid convalescent (MC). A two-way hierarchical cluster analyses was performed on differential IgG responses to the 33 antigens in 3 patients with AI, EC and MC sera, and 4 patients with only AI and EC sera (Figure S3). A one tailed t-test was carried out to look for differences where the EC antigen intensity is significantly greater than the AI antigen intensity, possibly indicating the selection of a protective antibody response. We observed a significant increase in the IgG response from AI to EC in the following antigens, which are ranked according to their p-values: Apc5 (2) (1.12E-03), Drs23 (3) (1.23E-03), Vps62 (1.57E-03), Rad50 (1.83E-03), Ssu1 (3.17E-03), Yor1 (3) (5.33E-03), Ipf885 (5.33E-03), Pga4 (5.88E-03), Cdr4 (3) (7.22E-03), Cfl91 (2) (0.0231), Cyr1 (2) (0.0274), Ipf25023 (2) (0.0330), Gsl2 (2) (0.0374), Chs1 (2) (0.0393), and Snq2 (3) (0.0486). The identified antigens could potentially be efficacious vaccine candidates due to the fact that the IgG response is being positively selected over the course of infection.

## Discussion

In this study, we have developed a *C. albicans* cell surface protein microarray and profiled host humoral responses during conmmensal colonization and during the progression of candidemia. Thirteen novel serodiagnostic antigens were identified for differentiating acute candidemia from commensal sensitization and 33 antigens were found to discriminate convalescent candidemia from non-candidemia controls. The sensitivity and specificity for the identification of acute candidemia determined by the top 10 antigens from the set of 13 serodiagnostic markers are comparable to that obtained using the method of 2D-PAGE and immunoblots [17]. When using the top 5 antigens from the set of 33, both sensitivity and specificity are dramatically improved for convalescent candidemia. Pitarch *et al.* reported that the anti-Bgl2p IgG antibody levels mainly define the proteomic signature for candidemia patients [17]. In this study, Bgl2 is on the list of 33 diagnostic antigens from convalescent sera. Although it is classified as a serodominant antigen by acute candidemia sera, the BH-adjusted p-value of Bgl2 (0.116) is just above cutoff (0.05) to be considered as diagnostic by our definition, and the mean anti-Bgl2 antibodies in acute candidemia is higher than the mean in non-candidemia controls. Bgl2 is a glycoprotein and the glycan moieties on other b-1,3-glucanosyltransferases seem to contribute to antigenicity. Since our Bgl2 is expressed *in vitro* without any glycosylation, its antigenicity is likely different from the Bgl2 produced by *C. albicans* used in the 2D-PAGE immunoblots. The previously identified immunogenic heat shock protein 90 (Hsp90) is also one of 33 biomarkers for convalescent candidemia identified from this study. Hsp90 has been shown to elicit a protective humoral response [42],[43] and its antibodies are known to associate with patients that recover from candidiasis. The use of protein microarray technology allowed us to identify new diagnostic antigens that were missed by previous studies. The use of 2-D PAGE to accurately identify and separate clinical markers of candidemia from commensal sensitization is limited by the range in protein abundance and various properties associated with peptides such as their mass, isoelectric point, hydrophobicity, and post-translational modification, as well as the semi-quantitative nature of a Western [18]. Using a *C. albicans* cell surface protein microarray helped us overcome many of the technical difficulties found with traditional proteomics, since the expression level of recombinant-derived proteins vary by only a single log and the use of fluorescent-labeled antibodies allows for greater linearity, precision, and sensitivity in the quantitative measurement of the humoral response to *C. albicans*. One of the most beneficial aspects in the use of the protein microarray assay is its ability to detect significant differences in the IgG response that under traditional immunoblot conditions would be below the detectable threshold. However, a potential limitation to our study is that the microarray is based on recombinant peptides. Because of the cell free nature of our *in vitro* translated peptides, potential epitopes may have been lost due to miss folding and a lack of glycosylation, both of which may affect the conformational structure of the native protein. On the other hand, the removal of posttranslational modifications, such as glycosylation, from the peptides may have revealed hidden peptide epitopes only seen during a strong host immune response. A large collection of peptide epitopes may increase the specificity in diagnosis of infection. In support of this, our study has identified many new clinical biomarkers that are associated with differing states of interactions with the host as well as the characterization of potential new targets for therapeutics and vaccine candidates. To our knowledge, this is the first study using a protein microarray to analyze the serological response to an organism that is capable of existing as both commensal flora and an opportunistic pathogen in the human population.

Commensal colonization of *C. albicans* is common in humans and attenuated host immunity is a perquisite for the transition from commensal colonization to infection. Historically, it was believed that *C. albicans* switched from a commensal to a pathogen using distinct pathogen-associated genetic programs when the host immune status was altered. An intriguing review challenges this notion, Hube postulates that *C. albicans* exists in a permanent host-pathogen interplay where overgrowth and invasion is only observed under immunocompromising conditions[44]. The review puts forth two-models of a permanent infection strategy: (1) constitutive gene expression where attenuated immunity induces little or no change in the pathogenic profile of *C. albicans* or (2) a variable transcriptional profile where *C. albicans* expression is dependent on the stage- and tissue-specific interactions with the host. Our study indicates the existence of permanent host-pathogen interplay with variable gene expression over the course of infection. The serological response to the entire *C. albicans* cell surface protein microarray detected considerable homogeneity as well as differences in the patterns of antigens recognized among patients and healthy individuals. The majority of healthy individuals and uninfected hospital patients have moderate to strong IgG responses to many *C. albicans* cell surface proteins that have long been associated with virulence or hyphal-regulation (a hallmark of virulence in itself). In agreement with our protein microarray data, Naglik *et al.* observed similar levels of IgG titers to the hyphal wall protein Hwp1 in patients with oral candidiasis and asymptomatic mucosal infections as well as healthy culture-negative controls [23]. These serodominant cross-reactive antigens include adhesins such as Als1, Als3, Als5, Hwp1 and Int1 and hyphal-regulated genes such as Als3, Hwp1, Ece1, Hyr1, and Cdc24. Both functional groups are known to be important for invasion and virulence [45]. Among the identified serodominant antigens are many previously characterized immunogenic peptides such as Bgl2 [17], Tkl1 [15], Hwp1 [13],[23], Eft2 [15], and Cdc24 [13]. Intriguingly, the average signal intensities to the top-forty serodominant antigens are higher in the healthy individuals than the uninfected hospital patients and acute candidiasis patients (10,380 vs. 8,837 and 8,825, respectively). It is interesting to speculate whether the healthy individuals' IgG response limits colonization and overgrowth since many of the serodominant antigens are against adhesins. In particular is the strong humoral response to the integrin-like protein, Int1, which may play dual roles in limiting both intestinal colonization of the cecum and systemic invasion of deep tissue organs [46],[47]. Another interesting serodominant antibody response is to the protein Ece1, which has been shown to promote adhesion and is important for GI colonization[48]. *ECE1* transcription is highly expressed during GI colonization and invasion of host tissue [33],[48]. However, one can not discount that the high IgG titer of colonized individuals may be due to a previous superficial infections such candidal vaginitis [49],[50].

The microenvironmental conditions during commensal colonization of the host may also play a role in the induction of the IgG response to certain cell surface proteins. Previous studies have evaluated characteristics common to the GI and/or vulvovaginal tract such as blood, hypoxia, iron restriction and weak acid as modifiers of gene expression [9], [51]–[53]. Intriguingly, the expressions of these genes share common features to the identified serodominant antibodies. Interestingly, genes transcriptionally up-regulated in blood (Als1, Als3, Hwp1, Ece1, Hyr1, and Bgl2) were serodominant and cross-reactive with both positive and negative candidiasis individuals, as were genes up-regulated under hypoxic conditions (Als1, Als3, Hwp1, Rbt5, Utr2, and Tos1), iron restriction (Int1, Rbt5, and Fet35), and weak acid (Crp1, Fet35, and Ipf9655) (Table 1). Furthermore, some of the serodominant antigens (i.e. Als3, Ece1, Hwp1, and Rbt5) have been shown to be induced during the invasion of epithelial or endothelial cells [11],[33]. Therefore, the expression of the serodominant antigens in healthy individuals indicates the existence of permanent host-pathogen interplay during commensal colonization. In addition, the presence of serodominant IgGs in all 50 healthy individuals suggests that commensal colonization is much more prevalent than previously reported.

One of the most challenging tasks in characterizing serodiagnostic antigens from *C. albicans* is the identification of discriminating peptides that can differentiate between commensal colonization and candidemia with high sensitivity and specificity. By profiling antibody response from patients with varying stages of candidemia against healthy individuals and candidemia-negative hospital patients, we have identified 13 diagnostic antigens for acute phase of candidemia and 33 for the early/mid convalescent candidemia. The serologic signature in candidemia patients likely reflects an alteration in the level of those proteins due to a change either in transcription and/or protein stability. Stage- and tissue-specific gene expression during the course of systemic infection is expected as *C. albicans* cells transition through differing microenvironments of the host. Among the 13 diagnostic antigens for acute candidemia, three are associated with drug resistance (Cdr1, Cdr4, and Yor1) [36]. The exposure to antifungal drugs in patients undergoing acute candidemia may have acted as an additional environmental stress that stimulates the expression of these antifungal drug transporters [54]. Intriguingly, two out of the 13 biomarkers are the osmosensors Sln1 and Nik1 for the HOG pathway that is responsible for osmotic and oxidative stress adaptation in *C. albicans* [34],[35]. The host-pathogen interaction commonly associated with oxidative stress is typically seen during phagocytosis by neutrophils, the initiating immune response to *C. albicans* overgrowth and infection. Furthermore, a study of global transcriptional responses to oxidative stress observed an increase in the transcriptional expression of *CDR4* (4.1-fold), *RAS2* (2.5-fold) and *ALS9* (1.5-fold) [35]. Taken together, our data indicates a strong correlation between the IgG response to oxidative stress-related cell surface proteins and the initial cell-mediated immune response during acute candidemia. In further agreement, previous studies have shown that oxidative stress functions are primarily induced when *C. albicans* is initially exposed to human blood or following phagocytosis by neutrophils and granulocytes [7],[9],[10],[55]. The 33 convalescent diagnostic antigens include proteins involved in iron acquisition, cell wall biogenesis, membrane lipid organization, and drug resistance. Of particular interest is the dramatic increase in antibodies to proteins for iron acquisition (Cfl91, ferric reductase; Rbt5 and Csa1, hemoglobin receptors; and Flc1, heme uptake). Iron is an essential nutrient for *C. albicans*. Circulating iron in serum is bound to transferrin and ferric reductases are required in the acquisition of iron from transferrin. Interestingly, Cfl91 is found as a biomarker for both acute and convalescent candidemia patients. Of particular interest is the increase antibody response to hemoglobin and heme-related proteins as these molecules are normally sequestered in erythrocytes [56]. The proteins Rbt5, Csa1 and Flc1 are required for iron acquisition from hemoglobin or heme [39],[40] and are diagnostic antigens only for convalescent candidemia. Thus, it is interesting to speculate whether free hemoglobin becomes a by-product of lysed erythrocytes after post-operative surgery or other invasive clinical procedures. Nevertheless, the data from this study should provide critical information for the development of diagnostic antigenic profiles for patients at risk for candidemia and for the assessment of progression of hematogenously disseminated candidiasis. Future studies will need to be done to determine whether serological differences exist between superficial and systemic infections, as well as commensal sensitization.

The development of the antigenic profiles over the course of candidiasis (acute infection, early convalescence, and mid convalescence) may also provide insight into a protective humoral response against *C. albicans*. Even though previous sensitization to commensal colonization does not limit mortality or even morbidity in patients, experimental studies have identified protective antibodies against hematogenously disseminated candidiasis, such as heat shock protein 90 (Hsp90) or β-mannan [57]–[60]. Future studies will need to address whether the serodiagnostic antigens identified in this study could provide protection from hematogenously disseminated candidiasis. Of particular interest are the convalescent serodiagnostic antigens where the EC antigen intensity is significantly greater than the AI antigen intensity, which may possibly indicate the selection of a protective antibody response.

## Materials and Methods

### Ethics statement

Human sera from candidemia patients and hospitalized patients were collected from SH-UF under protocols approved and created by the UF Institutional Review Board. Sera from healthy individuals were obtained from volunteers at the General Clinical Research Center at the University of California, Irvine. Written, informed consent was obtained from participants.

### Collection of candidemia and control sera

Candidemia was defined as the recovery of *C. albicans* from blood cultures. Sera from candidemia patients and hospitalized patients (no clinical or microbiological evidence of candidemia) were collected from SH-UF as previously published [61]. Briefly, patients at SH-UF were identified on the day blood cultures were positive for *C. albicans*. The Infectious Diseases Consultation Service at SH-UF identified controls. Sera were collected and stored at −70°C in the repository at the UF Mycology Research Unit. For patients with candidemia, sera were obtained from the earliest possible date on or after the date that the first positive cultures were drawn. In all cases, this was within 7 days of the first positive culture (acute-phase sera). For ten patients with candidemia, sera were also recovered 4 to 12 weeks after the date on which the first positive cultures were drawn (convalescent-phase sera).

### Microarray construction and antibody profiling

Cell surface proteins were selected from the *Candida* Genome Database (CGD) using keywords such as “cell surface”, “plasma membrane”, and “cell wall”. The CGD annotation of cell surface proteins is based on published experiments [32], [62]–[66], function-based prediction of cellular localization, and sequence prediction. Known antigenic proteins are also included as controls (Bgl2, Eno1, Pgk1, Gap1, Cdc19, Tkl1, Hsp90, and members of the Hsp70 family) [15],[17]. Coding regions of the genes were PCR amplified from the clinical isolate SC5314 of *C. albicans* with primers listed in Table S2, and cloned into a pXT7 expression vector with a HA-tag at the N-terminus and His-tag at the C-terminus by homologous recombination in *E. coli* as described [67]. Protein expression was carried out using an *E. coli* based cell-free *in vitro* transcription/translation system (RTS 100 *E. coli* HY kit, Roche). The protein microarray was made by printing the peptides onto nitrocellulose-coated FAST glass slides (Schleicher & Schuell) using the OmniGrid 100 (GeneMachines) in the UCI Microarray Facility. Each peptide was printed in duplicate and showed homogenous spot morphology as well as low background. Internal controls consisting of buffer alone and a reaction mixture with no DNA were also printed onto the FAST slides. After the addition of the plasma samples the microarray was incubated with a biotin-conjugated donkey anti-human IgG Fc_γ_ fragment specific secondary antibody (Jackson Immunoresearch). The secondary antibody was then removed and the microarray was incubated with Streptavidin: SureLight ® P-3 (Columbia Biosciences). Details concerning microarray construction and controls, antibody profiling, data normalization, as well as the reproducibility and validity of the microarray are given in the Text S1.

### Statistical analysis

All analysis was performed using the R statistical environment (http://www.r-project.org). It has been noted in the literature that data derived from microarray platforms is heteroskedatic [68]–[70]. This mean-variance dependence has been observed in the arrays presented in this manuscript [71],[72]. In order to stabilize the variance, the vsn method [73] implemented as part of the Bioconductor suite (www.bioconductor.org) was applied to the quantified array intensities. In addition to removing heteroskedacity, this procedure corrects for non-specific noise effects by finding maximum likelihood shifting and scaling parameters for each array such that the variances of a large number (default setting used: 85%) of the spots on the array are minimized. In other words, the method assumes that variance in binding for the vast majority of the proteins on the array are due to noise rather than true differential immunological response. In essence, 85% of the spots on the array are used as controls for sample-by-sample normalization. This calibration method has been shown to be effective on a number of platforms [74]–[76]. A simple ranking normalization where all of the proteins are ordered for each sample by binding intensity and assigning the integer rank was performed as well with similar results (results not shown). Finally, VSN normalized data is retransformed with the ‘sinh’ function to allow visualization and discussion at an approximate raw scale.

Diagnostic biomarkers between groups were determined using a Bayes regularized t-test adapted from Cyber-T for protein arrays [69],[77]. To account for multiple testing conditions, the Benjamini and Hochberg (BH) method was used to control the false discovery rate [78]. Statistical analyses were performed with R 2.0 (www.r-project.org) and STATA (version 10.0, StataCorp). Multiple antigen classifiers were constructed using linear and non-linear Support Vector Machines (SVMs) using the “e1071” R package. To prevent overfitting and show the generalization of the classification method, 10 repeats of three-fold cross-validation were performed. In this methodology, the data is split into 3 class-stratified subsets. For each subset, a classifier is trained using the remaining two-thirds of the data. The classifier is then evaluated on the one-third of the data not used for training. This process is repeated for each split and for 10 different splits, yielding 30 evaluation measures. The ROCR package was used to construct receiver-operating-characteristic curves and perform sensitivity and specificity analyses. Blast2Go (www.blast2go.org) was used for gene ontology annotation and enrichment analysis. To confirm that the identified antigens were accurate, their vectors were resequenced. The Tables S3 and S4 list the statistical data of acute and convalescent candidemia patients, respectively.

### Accession numbers

Detailed information for the genes/proteins from this study can be found at the Candida Genome Database http://www.candidagenome.org. The gene names and ORF numbers are listed here: *INT1* (19.4257), *CWH41* (19.4421), *PGA13* (19.6420), *RBT5* (19.5636), *HWP1* (19.1321), *SLK19* (19.6763), *YPS7* (19.6481), *ALS3* (19.1816), *CHS2* (19.7298), *EFT2* (19.5788), *IPF9655* (19.3988), *GNP3* (19.7565), *PHR3* (19.5632), *ECE1* (19.3374), *BGL2* (19.4565), *PAN1* (19.19.886), *OSH2* (19.5095), *CRP1* (19.4784), *PRY1* (19.2787), *PGA60* (19.5588), *UTR2* (19.1671), *HNM1* (19.2003), *HYR1* (19.4975), *WSC4* (19.7251), *CDC24* (19.3174), *HYR3* (19.575), *DNF2* (19.932), *MEP2* (19.5672), *GCA1* (19.4899), *CWH43* (19.3225), *FRE10* (19.1415), *ALS5* (19.5736), *ALS1* (19.5741), *SLN1* (19.3256), *FCY21* (19.1357), *TOS1* (19.1690), *FET34* (19.4215), *TKL1* (19.5112), *CDR1* (19.6000), *CFL91* (19.1844), *CDR4* (19.5079), *ALS9* (19.5742), *CDC19* (19.3575), *NIK1* (19.5181), *CHS8* (19.5384), *RTA4* (19.6595), *TRK1* (19.600), *YOR1* (19.1783), *CSC25* (19.6926), *RAS2* (19.5902), *DRS23* (19.323), *IPF25023* (19.2296), *ALS6* (19.7414), *VPS62* (19.1800), *SNQ2* (19.5759), *IPF885* (19.7214), *CAG1* (19.4015), *HNM4* (19.2946), *APC5* (19.6861), *HSP90* (19.6515), *CSA1* (19.7114), *GSL2* (19.3269), *PGA4* (19.4035), *FLC1* (19.2501), *CHS1* (19.7298), *IPF22247* (19.4940), *YCK22* (19.2222), *SSU1* (19.7313), *RAD50* (19.1648), and *CYR1* (19.5148).

## Supporting Information

Text S1 Supplemental Experimental Procedures and Supplemental References
(0.08 MB DOC)

Figure S1 *C. albicans* cell surface protein microarray. Representative image of the cell surface protein microarray of *C. albicans* hybridized with the sera of an acute candidemia patient. The array consisted of sixteen subsets. Each of the *C. albicans* cell surface peptides were printed in duplicate. The yellow box indicates a duplicated print of buffer alone and the red box shows a duplicate print of reaction mixture with no DNA.
(0.13 MB PDF)

Figure S2 Global expression profile of *C. albicans* cell surface antigens. Heatmap of the entire *C. albicans* cell surface protein microarray probed with a collection of acute candidemia patients (n = 18), early and mid convalescent candidemia patients (n = 10), uninfected hospital patients (n = 12) and healthy individuals (n = 50). The antigens are in columns and are sorted by normalized mean intensity. The colorized scale ranks the antigens with red being the strongest, bright green the weakest, and black in between.
(0.22 MB PDF)

Figure S3 Development of the antigenic profile overtime in candidiasis patients. Two-way hierarchical cluster analyses of differential IgG response to the 33 convalescent serodiagnostic antigens (rows) and serum specimens (columns) from candidemia patients. The patients are ordered from left to right starting with the acute infection (AI) phase, early convalescent (EC), and mid convalescent (MC). The colorized scale ranks the antigens with red being the strongest, bright green the weakest, and black in between. Cell surface proteins that showed a significant increase in IgG response from AI to EC are labeled red (p-value ≤0.05).
(0.17 MB PDF)

Table S1 Study population characteristics
(0.03 MB PDF)

Table S2 List of proteins and primer sequences on microarray
(0.22 MB XLS)

Table S3 Statistical data of acute candidemia patients
(0.24 MB XLS)

Table S4 Statistical data of convalescent candidemia patients
(0.28 MB XLS)
