# Correction: Serological Profiling of a *Candida albicans* Protein Microarray Reveals Permanent Host-Pathogen Interplay and Stage-Specific Responses during Candidemia

**DOI:** 10.1371/annotation/eff399e1-51e0-43b1-bdf4-1a11e9ada9bd

**Published:** 2010-09-30

**Authors:** A. Brian Mochon, Ye Jin, Matthew A. Kayala, John R. Wingard, Cornelius J. Clancy, M. Hong Nguyen, Philip Felgner, Pierre Baldi, Haoping Liu

The second author's name was written incorrectly. The correct name is: Ye Jin. The correct citation is: Mochon AB, Jin Y, Kayala MA, Wingard JR, Clancy CJ, et al. (2010) Serological Profiling of a Candida albicans Protein Microarray Reveals Permanent Host-Pathogen Interplay and Stage-Specific Responses during Candidemia. PLoS Pathog 6(3): e1000827. doi:10.1371/journal.ppat.1000827. In the author contributions, "Performed the experiments" should read: ABM YJ. 

