# Supplementary material for: Serological Profiling of a Candida albicans Protein Microarray Reveals Permanent Host-Pathogen Interplay and Stage-Specific Responses during Candidemia
Source: PLoS Pathog. 2010 Mar 26;6(3):e1000827. doi: 10.1371/journal.ppat.1000827 (PMC2845659; doi:10.1371/journal.ppat.1000827)
Supplement: Text S1 — Supplemental Experimental Procedures and Supplemental References (0.08 MB DOC) [file ppat.1000827.s001.doc]

Supporting Information

**Serological Profiling of a *Candida* *albicans* Protein Array Reveals Permanent Host-Pathogen Interplay & Stage-Specific Responses during Candidemia**

A. Brian Mochon, Jin Ye, ****Matthew A. Kayala****, John R. Wingard, ****Cornelius J. Clancy, M. Hong Nguyen, Philip Felgner,**** ****Pierre Baldi, and Haoping Liu****

Supplemental Experimental Procedures

***Candida albicans* cell surface protein microarray construction and hybridization**

Coding regions of the genes were PCR amplified from the clinical isolate SC5314 of *C. albicans* and cloned into a pXT7 expression vector with a HA-tag at the N-terminus and His-tag at the C-terminus by homologous recombination in *E. coli*. Protein expression was carried out using an *E. coli* based cell-free *in vitro* transcription/translation system (RTS 100 *E. coli* HY kit, Roche). The collection contains 451 His- or HA-tagged peptides that represent 363 different proteins since ORFs >3,000 bps were cloned into two or more segments. All tagged proteins were confirmed individually by western blot and again on the protein microarray. Figure S1 shows a representative image of the microarray hybridized with the serum of an acute candidemia patient. Each peptide was printed in duplicate and showed homogenous spot morphology as well as low background. The mean correlation between the duplicate printings of each peptide was 0.829. Internal controls were also printed onto the nitrocellulose pad consisting of buffer alone and a reaction mixture with no DNA. Monoclonal antibodies against the His- and HA- conjugation were employed as predictors of protein expression and provided 98% expression efficiency on the proteome array. All hybridizations were done under the same conditions and dilutions.

**PCR amplification of genes encoding *C. albicans* cell surface proteins**

To search for genes encoding *C. albicans* cell surface proteins, we mined the Candida Genome Database ([www.candidagenome.org](http://www.candidagenome.org/)). The annotations are based on publications from several groups [1-6]. A high throughput *in vivo* transformation system using a pXT7 linear vector [7] was employed for cloning PCR products of genes encoding *C. albicans* cell surface proteins. For PCR amplification, all forward and reverse primers have a common 33 nucleotide-long sequence at the 5’ end, followed by a gene-specific sequence (20-26 nucleotides). The common 33-nucleotide-long sequences at the 5’ end are homologous with the end sequences on the pXT7 linear vector. Each ORF was amplified from genomic DNA by using gene-specific primers. For genes larger than 3,000kb, multiple sets of primers were employed to sequence multiple segments of the gene to reduce the difficulty of PCR amplification. Table S2 lists the cell surface proteins and their corresponding primer sequences that were printed onto the protein microarray.

# *In vivo* recombination and *in vitro* transcription/translation

For *in vivo* recombination, we employed a previously reported high-efficiency (>90%) method [7]. Briefly, an equal molar amount of pTX7 linear vector and each of the PCR products were mixed and incubated with 50μl of DH5α competent cells on ice for 60 minutes. After a 1 minute-heat shock at 42°C, the mixture was chilled for 1 minute, and then incubated in 250μl of SOC (super optimal catabolizer) medium for 1 hour. Afterwards, the mixture was mixed with 1.5ml of LB medium supplemented with 50μg/ml kanamycin and then grown overnight at 37°C. Plasmids with ORF inserts were then isolated using QIAprep 96 Turbo Miniprep Kit (Quiagen, CA). The resulting circular plasmids with gene inserts were subjected to *in vitro* transcription-translation using the RTS 100 *E. coli* HY kit (Roche). An aliquot of the plasmid was mixed with cell-free *in vitro* transcription/translation reagents and incubated at 30°C for 6 hours. The quantity and quality of expressed proteins were assessed by western blots. The blots were probed with anti-poly-His mAb (Sigma) followed by alkaline phosphatase-conjugated goat anti-mouse IgG (H+L) (BioRad) or rat peroxidase-conjugated anti-HA Ab (Roche).

# Protein microarray construction

The *in vitro* expressed proteins were printed in duplicate onto nitrocellulose-coated FAST glass slides (Schleicher & Schuell) at a density of 960 spots per slide using an automatic GMS417 robot (Genetic Microsystems). Proteins positive for either HA-tag or His-tag were immobilized on the array chips (HA-positive: 84%; His-positive: 61%). Proteins having at least one of the tags provided 98% coverage of the *in vitro* expressed proteins. For microarray detection, the proteome chips were blocked for 30 minutes with protein array blocking buffer (Schleicher & Schuell). Concurrently, a 1/50 dilution of human serum was incubated with 10% *E. coli* lysate in protein array blocking buffer for 30 minutes at room temperature to remove antibodies directed against *E. coli* antigens. The protein microarray was then probed with the human serum at 4°C overnight. The serum was aspirated and washed 3 times with TTBS. The protein array was then incubated with a biotin-conjugated donkey anti-human IgG Fc_γ_ fragment specific secondary antibody (Jackson Immunoresearch) for 2 hours at room temperature. The secondary antibody was then aspirated and washed 3x with TTBS and then incubated with Streptavidin: SureLight ® P-3 (Columbia Biosciences). GSI Lumonics scanned the hybridized slides and the intensity for each cell surface peptide was determined. Within each protein microarray, nine pairs of RTS reagents were printed and used as internal controls for non-specific binding. The scanning of each patient was evaluated using a fixed titer of sera and were later adjusted using LP and PMT to obtain a relative level of intensity using spots without DNA and with buffer alone.

# Supplemental References

1. Fan J, Chaturvedi V, Shen SH (2002) Identification and phylogenetic analysis of a glucose transporter gene family from the human pathogenic yeast Candida albicans. J Mol Evol 55: 336-346.

2. Monteoliva L, Matas ML, Gil C, Nombela C, Pla J (2002) Large-scale identification of putative exported proteins in Candida albicans by genetic selection. Eukaryot Cell 1: 514-525.

3. De Groot PW, Hellingwerf KJ, Klis FM (2003) Genome-wide identification of fungal GPI proteins. Yeast 20: 781-796.

4. Urban C, Sohn K, Lottspeich F, Brunner H, Rupp S (2003) Identification of cell surface determinants in Candida albicans reveals Tsa1p, a protein differentially localized in the cell. FEBS Lett 544: 228-235.

5. Alberti-Segui C, Morales AJ, Xing H, Kessler MM, Willins DA, et al. (2004) Identification of potential cell-surface proteins in Candida albicans and investigation of the role of a putative cell-surface glycosidase in adhesion and virulence. Yeast 21: 285-302.

6. Braun BR, van Het Hoog M, d'Enfert C, Martchenko M, Dungan J, et al. (2005) A human-curated annotation of the Candida albicans genome. PLoS Genet 1: 36-57.

7. Davies DH, Liang X, Hernandez JE, Randall A, Hirst S, et al. (2005) Profiling the humoral immune response to infection by using proteome microarrays: high-throughput vaccine and diagnostic antigen discovery. Proc Natl Acad Sci U S A 102: 547-552.
