# Supplementary figures and images for: Serological Profiling of a Candida albicans Protein Microarray Reveals Permanent Host-Pathogen Interplay and Stage-Specific Responses during Candidemia
Source: PLoS Pathog. 2010 Mar 26;6(3):e1000827. doi: 10.1371/journal.ppat.1000827 (PMC2845659; doi:10.1371/journal.ppat.1000827)

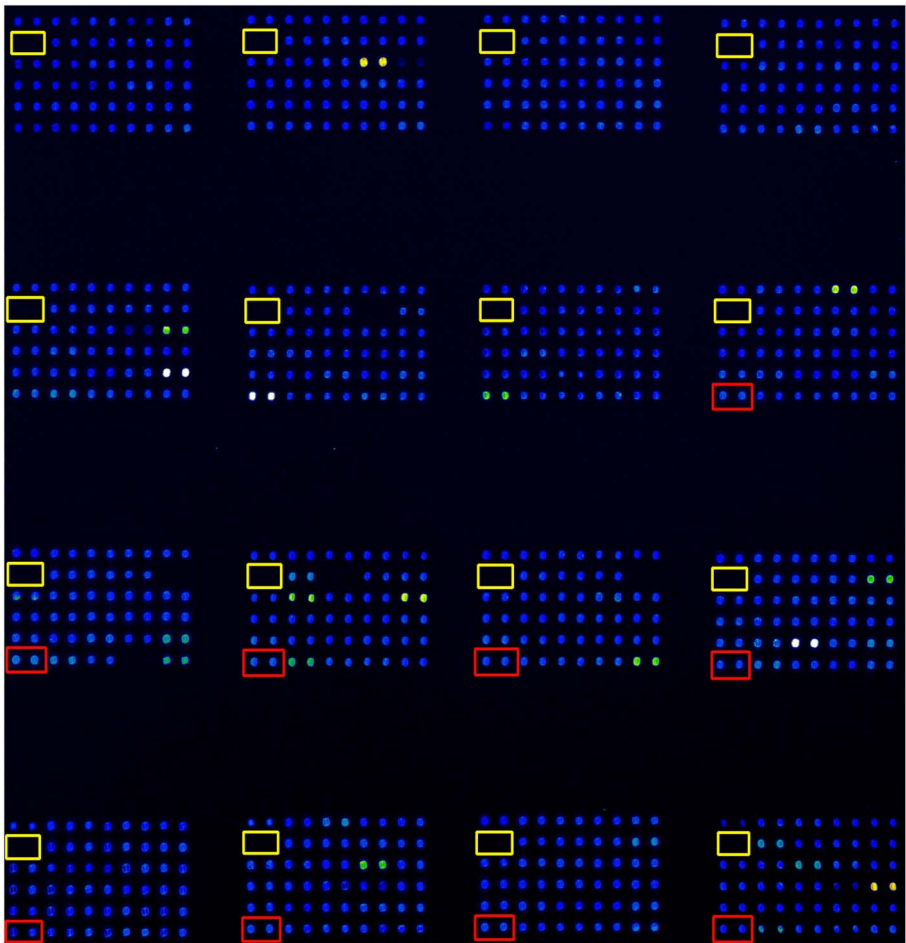

Supplement: Figure S1 — C. albicans cell surface protein microarray. Representative image of the cell surface protein microarray of C. albicans hybridized with the sera of an acute candidemia patient. The array consisted of sixteen subsets. Each of the C. albicans cell surface peptides were printed in duplicate. The yellow box indicates a duplicated print of buffer alone and the red box shows a duplicate print of reaction mixture with no DNA. (0.13 MB PDF) [file ppat.1000827.s002.pdf]

Color Key and Histogram

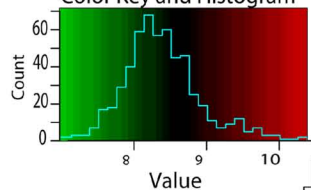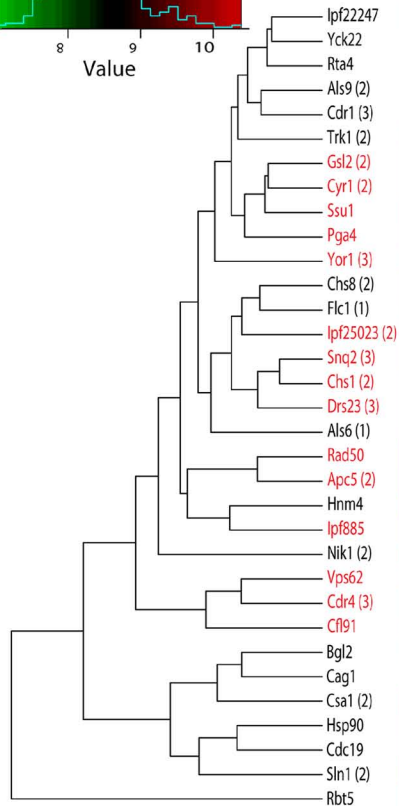

Candidemia patients:

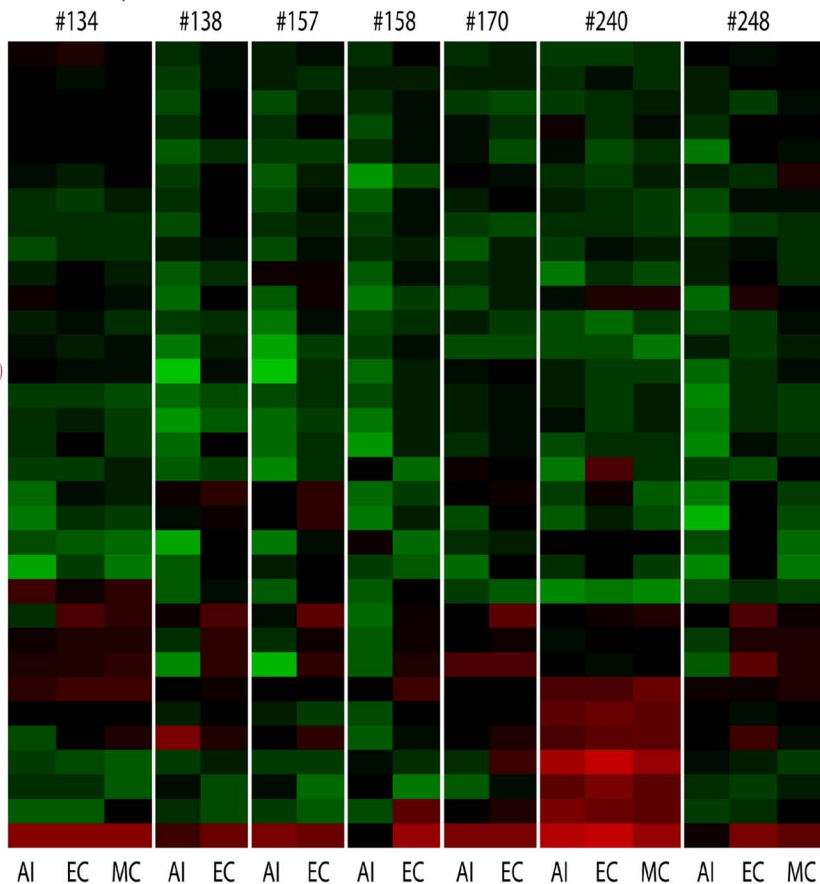

Supplement: Figure S3 — Development of the antigenic profile overtime in candidiasis patients. Two-way hierarchical cluster analyses of differential IgG response to the 33 convalescent serodiagnostic antigens (rows) and serum specimens (columns) from candidemia patients. The patients are ordered from left to right starting with the acute infection (AI) phase, early convalescent (EC), and mid convalescent (MC). The colorized scale ranks the antigens with red being the strongest, bright green the weakest, and black in between. Cell surface proteins that showed a significant increase in IgG response from AI to EC are labeled red (p-value ≤0.05). (0.17 MB PDF) [file ppat.1000827.s004.pdf]
