# Supplementary material for: Serological Profiling of a Candida albicans Protein Microarray Reveals Permanent Host-Pathogen Interplay and Stage-Specific Responses during Candidemia
Source: PLoS Pathog. 2010 Mar 26;6(3):e1000827. doi: 10.1371/journal.ppat.1000827 (PMC2845659; doi:10.1371/journal.ppat.1000827)
Supplement: Figure S2 — Global expression profile of C. albicans cell surface antigens. Heatmap of the entire C. albicans cell surface protein microarray probed with a collection of acute candidemia patients (n = 18), early and mid convalescent candidemia patients (n = 10), uninfected hospital patients (n = 12) and healthy individuals (n = 50). The antigens are in columns and are sorted by normalized mean intensity. The colorized scale ranks the antigens with red being the strongest, bright green the weakest, and black in between. (0.22 MB PDF) [file ppat.1000827.s003.pdf]

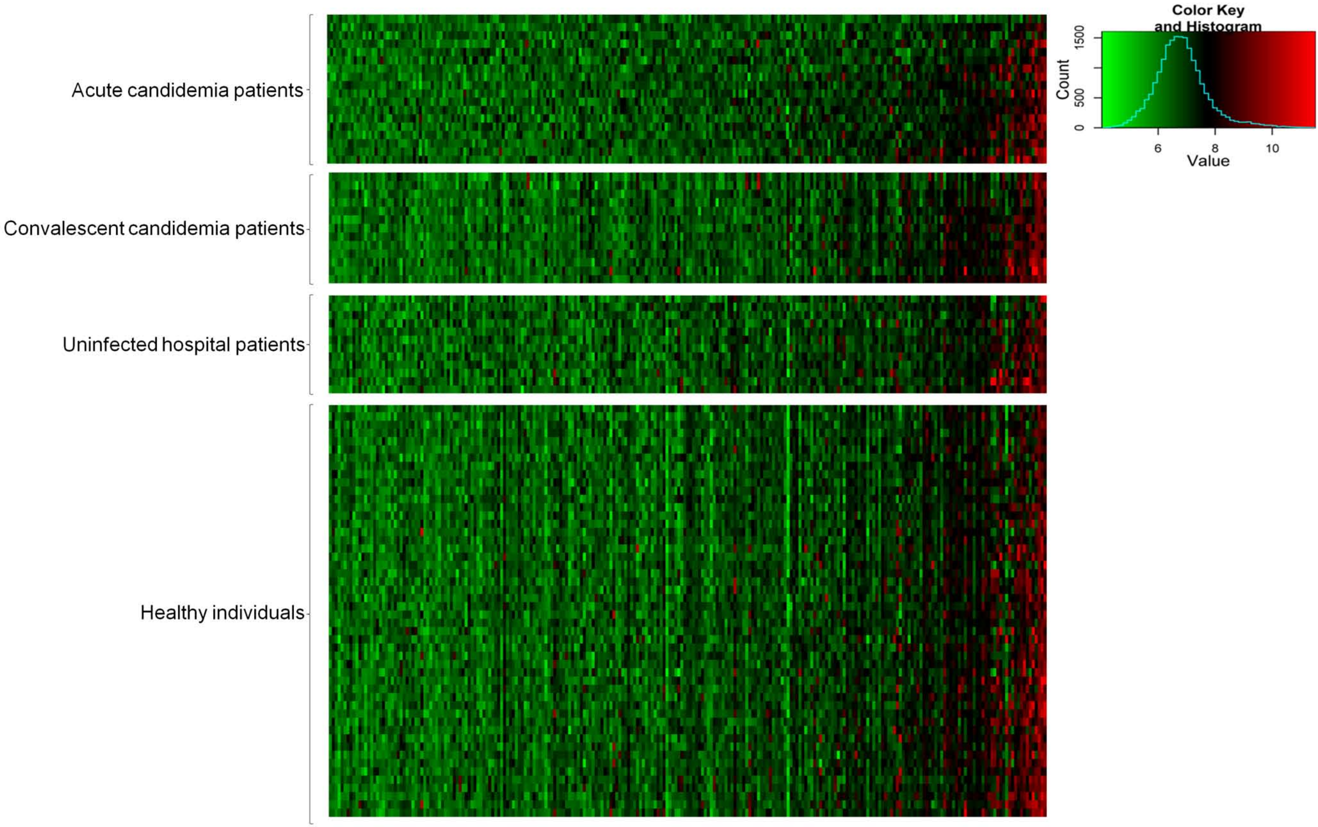

Acute candidemia patients
Convalescent candidemia patients
Uninfected hospital patients
Healthy individuals
Color Key
and Histogram
Count
1500
1000
500
0
6
8
10
Value
