# Supplementary material for: Serological Profiling of a Candida albicans Protein Microarray Reveals Permanent Host-Pathogen Interplay and Stage-Specific Responses during Candidemia
Source: PLoS Pathog. 2010 Mar 26;6(3):e1000827. doi: 10.1371/journal.ppat.1000827 (PMC2845659; doi:10.1371/journal.ppat.1000827)
Supplement: Table S1 — Study population characteristics (0.03 MB PDF) [file ppat.1000827.s005.pdf]

Table S1. Study population characteristics

Characteristics	Candidemia patients	Negative controls	
	C. albicans (n=21)	Hospital patients[4] (n=12)	Healthy subjects (n=50)
**Demographic factors**			
Median age (range)	50 (10 - 81)	---	35 (20 - 59)
**Gender**			
Male	16	---	14
Female	5	---	36
**Underlying diseases**			
Burn	7	---	---
Chronic TPN[1]	3	---	---
Intestinal surgery	2	---	---
Vascular surgery	2	---	---
Trauma	2	---	---
Immunosuppression[2]	2	---	---
Others[3]	3	---	---
**Portal of entry**			
Catheter-associated fungemia	13	---	---
Burn wound	4	---	---
Intestinal source	3	---	---
Endovascular infection	1	---	---
**Antifungal received**			
Fluconazole	13	---	---
Caspofungin	6	---	---
Voriconazole	1	---	---
Nystatin	1	---	---
**Outcome of hospital stay**			
Discharge	17	---	---
Death	4	---	---

[1] TPN: total parenteral nutrition

[2] Lymphoma and neutropenia; Crohns disease and primary immunodeficiency disorder (*n*=one each)

[3] Others include one each of myocardial infarction requiring an intra-aortic ballon pump, necrotizing pancreatitis with pseudocyst formation, and multiple sclerosis.

[4] Sera from uninfected hospital patients were collected from the surgical intensive care unit
